# Clinical mutational profiling and categorization of *BRAF* mutations in melanomas using next generation sequencing

**DOI:** 10.1186/s12885-019-5864-1

**Published:** 2019-07-05

**Authors:** Parvez M. Lokhandwala, Li-Hui Tseng, Erika Rodriguez, Gang Zheng, Aparna Pallavajjalla, Christopher D. Gocke, James R. Eshleman, Ming-Tseh Lin

**Affiliations:** 1 Department of Pathology, Johns Hopkins University School of Medicine, Johns Hopkins University School of Medicine, 1812 Ashland Ave, Suite 200, Baltimore, MD 21205 USA; 2 Department of Medical Genetics, National Taiwan University Hospital, Taipei, Taiwan; 3 Departments of Oncology, Johns Hopkins University School of Medicine, Johns Hopkins Hospital, Baltimore, MD USA

**Keywords:** *BRAF*, *NRAS*, Melanoma, Kinase-impaired, Categorization

## Abstract

**Background:**

Analysis of melanomas for actionable mutations has become the standard of care. Recently, a classification scheme has been proposed that categorizes *BRAF* mutations based on their mechanisms for activation of the MAPK pathway.

**Methods:**

In this analysis *BRAF*, *KIT*, *NRAS*, and *PIK3CA* mutations were examined by next generation sequencing (NGS) in 446 melanomas in a clinical diagnostic setting. *KRAS* and *HRAS* were also analyzed to elucidate coexisting *BRAF* and *RAS* mutations. *BRAF* mutations were categorized into class-1 (kinase-activated, codon 600), class-2 (kinase-activated, non-codon 600) and class-3 (kinase-impaired), based on the newly proposed classification scheme.

**Results:**

NGS demonstrated high analytic sensitivity. Among 355 mutations detected, variant allele frequencies were 2–5% in 21 (5.9%) mutations and 2–10% in 47 (13%) mutations. Mutations were detected in *BRAF* (42%), *NRAS* (25%), *KIT* (4.9%) and *PIK3CA* (2.7%). The incidence of class-1, class-2 and class-3 mutations were 33% (26% p.V600E and 6.1% p.V600K), 3.1 and 4.9% respectively. With a broader reportable range of NGS, class-1, class-2 and class-3 mutations accounted for 77, 7.4 and 12% of all *BRAF* mutations. Class-3 mutations, commonly affecting codons 594, 466 and 467, showed a higher incidence of coexisting *RAS* mutations, consistent with their RAS-dependent signaling. Significant association with old age and primary tumors of head/neck/upper back suggest chronic solar damage as a contributing factor for melanomas harboring *BRAF* p.V600K or class-3 mutations.

**Conclusion:**

This study categorizes the range, frequency, coexisting driver mutations and clinical characteristics of the three classes of *BRAF* mutations in a large cohort of melanomas in a clinical diagnostic setting. Further prospective studies are warranted to elucidate the clinical outcomes and benefits of newly developed targeted therapy in melanoma patients carrying each class of *BRAF* mutation.

**Electronic supplementary material:**

The online version of this article (10.1186/s12885-019-5864-1) contains supplementary material, which is available to authorized users.

## Background

Integrated multiplatform analyses including whole exon sequencing and whole genome sequencing have uncovered the landscape of genomic alterations and important implications for prognosis and therapy in different subtypes of melanomas [1–4]. Most melanomas had potentially actionable mutations in the mitogen-activated protein kinase (MAPK) pathway and phosphatidylinositol 3-kinase (PI3K/AKT/mTOR) pathway. These included *BRAF* mutations affecting codon 600, *NRAS* mutations affecting codon 61 (and less frequently codons 12 and 13), and *KIT* mutations within exons 9 and 11. Monotherapy with BRAF inhibitors and combined therapy with *BRAF* inhibitors and MEK inhibitors have been approved by the Food and Drug Administration in the United States of America and other countries worldwide for targeted therapy of metastatic melanoma [5–10]. In *NRAS*-mutated advanced melanoma, a progression-free survival benefit was observed in patients treated with binimetinib (a MEK inhibitor) compared with dacarbazine [11]. Tyrosine kinase inhibitors (imatinib, nilotinib and dasatinib) have also shown benefits for melanoma patients with activating *KIT* mutations [12–14]. Mutation detection among these genes is recommended for standard of care targeted therapy in patients with metastatic melanoma.

Cobas 4800, a real-time polymerase chain reaction (PCR) mutational assay, was the first FDA-approved companion diagnostic test to detect the *BRAF* p.V600E mutation [15]. However, next generation sequencing (NGS) platforms have become popular in the clinical diagnostic setting for simultaneous mutational profiling of a panel of genes for melanomas and other cancers [16, 17]. Using NGS assays with a higher analytic sensitivity and a broader reportable range, a variety of *BRAF* mutations were detected within exon 11 and 15 [18–20]. We previously categorized these *BRAF* mutations based on their impact on the kinase activity (activated versus impaired) of the protein, and proposed potential clinical implications [17, 21].

More recently, *BRAF* mutations are further categorized by Yao et al. based on their mechanisms for activation of the MAPK pathway [22, 23]. This classification includes class-1 (constitutive active RAS-independent monomer with high BRAF kinase activity involving codon 600), class-2 (constitutive active RAS-independent dimers with high or intermediate *BRAF* kinase activity involving codons outside 600, including *BRAF* fusion mutants), and class-3 (low or no BRAF kinase activity) mutations. Pre-clinical and clinical studies have demonstrated distinct oncogeneic mechanisms for each class of *BRAF* mutation, which in turn might predict different therapeutic strategies [17, 23, 24]. Previous studies, prior to the recent categorization of 3 classes of *BRAF* mutation, have shown impact of non-V600 *BRAF* mutations on disease characteristics and clinical outcomes in melanomas and colorectal cancers [18, 25–27]. Furthermore, categorization of *BRAF* mutations in lung cancers, based on the classification scheme proposed by Yao et al. has shown that cancers with class-2 and class-3 mutations have a more aggressive course and less favorable prognosis [28].

In this study *BRAF*, *NRAS*, *KRAS*, *HRAS*, *PIK3CA* and *KIT* mutations were examined in melanomas. We demonstrated coexisting mutations of these driver genes in melanomas, and categorize *BRAF* mutations based on the new classification system to elucidate their association with clinical characteristics.

## Methods

### Materials

A total of 502 formalin-fixed paraffin-embedded (FFPE) specimens with a diagnosis of malignant melanoma (*n* = 500) or melanoma in situ (*n* = 2) were submitted to the Molecular Diagnostics Laboratory at The Johns Hopkins Hospital between August 2013 and November 2017. The assay failed in 26 specimens (5.2%). Two metastatic specimens with prior BRAF inhibitor treatment were excluded. This include one pleural effusion specimen carrying 49% *BRAF* p.V600E, 19% *NRAS* p.Q61K and 3.3% *NRAS* p.Q61R coexisting mutations. The remaining 474 specimens with NGS results were submitted from 457 tumors of 455 patients (Additional file 1: Table S1). Paired specimens were submitted from 19 patients, including 9 patients with primary and metastatic tumor specimens and 8 patients with 2 metastatic tumor specimens showing the same mutational status, and 2 patients with two primary tumor specimens showing different mutational status. Eleven of 457 tumors with an estimated tumor cellularity of less than 10% and no *BRAF*, *RAS*, *KIT* and *PIK3CA* mutations detected were excluded for analysis. Among the remaining 446 tumors, there were 20 primary and/or metastatic mucosal melanomas, 2 metastatic uveal melanomas, 286 primary, recurrent or metastatic cutaneous melanomas, and 138 metastatic melanomas of unknown origin or without information of the primary site. Estimated tumor cellularity of these 446 melanoma specimens ranged from less than 10% to nearly 100%. Accompanied hematoxylin and eosin stained slides were reviewed by a pathologist (MTL). FFPE tissues from 1 to 10 unstained, 5 or 10-μm-thick sections were macro-dissected using Pinpoint reagents according the manufacturer’s protocol (ZymoResearch, Orange, CA). DNA was purified using QIAmp DNA kit (Qiagen, Valencia, CA) as described previously [29]. Concentration of DNA was determined by Qubit 2.0 Fluorometer (Life Technologies, Carlsbad, California). The Johns Hopkins Medicine institutional review board granted approval to this study.

### Next generation sequencing (NGS)

NGS was conducted using AmpliSeq Cancer Hotspot Panel (v2) (Life Technologies, Carlsbad, California) for targeted multi-gene amplification as described previously [29]. Sequencing data of the targeted genes were analyzed using Torrent Suite (Life Technologies). Mutations were identified and annotated through both Torrent Variant Caller and direct visual inspection of the binary sequence alignment/map (BAM) file on the Broad Institute’s Integrative Genomics Viewer (IGV) [30]. Melanoma specimens were analyzed for *BRAF* (NM_004333), *KIT* (NM_000222), *NRAS* (NM_002524) and *PIK3CA* (NM_006218) genes for clinical reporting. *BRAF* mutations of the first 152 melanoma specimens were previously categorized based on their impact on the kinase activity [17]. *KRAS* (NM_033360) and *HRAS* (NM_005343) mutations were retrospectively analyzed to elucidate coexistence of *RAS* and kinase-impaired *BRAF* mutations. *GNAQ* (NM_002072) and *GNA11* (NM_002067) mutations were retrospectively analyzed for the uveal melanomas. During our validation of this NGS assay, a cutoff of background noise at 2% was chosen for single nucleotide variations [31]. *BRAF* mutations were categorized according to the recently proposed classification scheme [23]. These included class-1 (high kinase activity involving codon 600), class-2 (high or intermediate kinase activity involving codons outside 600), and class-3 (impaired kinase activity) mutations.

### Statistical analysis

Fisher exact test or **χ**^2^ test were performed to calculate *P* values.

## Results

### Mutational profiling of 446 melanomas

NGS revealed 118 (26%) of 446 melanomas with no mutation, 42% with *BRAF* mutations, 25% with *NRAS* mutations, 4.9% with *KIT* mutations, and 2.0, 2.7 and 2.7% with *HRAS*, *KRAS* and *PIK3CA* mutations, respectively (Table 1). Metastatic melanomas of unknown origin or without information of the primary site showed similar mutation rates to those of cutaneous melanomas, suggesting most of these cases were of cutaneous origin. Mucosal melanomas showed a higher incidence of no mutation (*P* < .01) and a lower incidence of *BRAF* mutations (*P* < .001), as compared to those of cutaneous melanomas. Analysis of the *GNA11* and *GNAQ* genes in two metastatic uveal melanomas revealed *GNA11* p.Q209L and *GNAQ* p.Q209P, respectively.

**Table 1:** Mutational profiling of 446 melanomas

Melanoma	Negative	*BRAF*	*HRAS*	*KRAS*	*NRAS*	*KIT*	*PIK3CA*
Uveal (n = 2)	2 (100%)	0 (0%)	0 (0%)	0 (0%)	0 (0%)	0 (0%)	0 (0%)
Mucosal (*n* = 20)	12 (60%)	1 (5.0%)	0 (0%)	1 (5.0%)	4 (20%)	3 (15%)	1 (5.0%)
Cutaneous (*n* = 286)^a^	72 (25%)	132 (46%)	9 (3.1%)	7 (2.4%)	64 (22%)	14 (4.9%)	6 (2.1%)
Others (*n* = 138)^b^	32 (23%)	56 (41%)	0 (0%)	4 (2.9%)	42 (30%)	5 (3.6%)	5 (3.6%)
Total (*n* = 446)^a^	118 (26%)	189 (42%)	9 (2.0%)	12 (2.7%)	110 (25%)	22 (4.9%)	12 (2.7%)

^a^11 specimens with no mutation detected and an estimated tumor cellularity of less than 10% were excluded

^b^Metastatic melanomas of unknown origin or without information of the primary site

### *BRAF* mutations

**Fig. 1:**
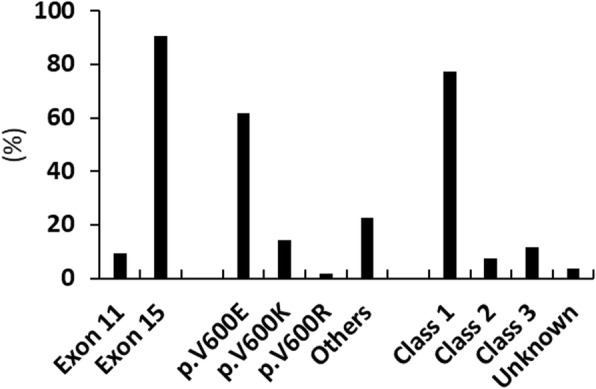
Distribution of *BRAF* mutations. *BRAF* mutations are categorized into class-1 (high kinase activity involving codon 600), class-2 (high or intermediate kinase activity involving codons outside 600), and class-3 (impaired BRAF kinase activity). [23]

A total of 30 unique *BRAF* mutations were detected in 189 *BRAF*-mutated melanomas, including one tumor with two *BRAF* mutations (Table 2). Eighteen (9%) mutations were located within exon 11 and 172 (91%) within exon 15. p.V600E (62%) and p.V600K (14%) were the two most common *BRAF* mutations (Fig. 1). Mutations occurring outside codon 600 were seen in 43 tumors (23% of *BRAF*-mutated melanomas or 9.6% of melanomas). There were 147 (77%) class-1 mutations, 14 (7.4%) class-2 or likely class-2 mutations, and 22 (12%) class-3 or likely class-3 mutations (impaired kinase activity) and 7 mutations of unknown class (3.7%) (Table 2 and Fig. 1). The overall incidence among this cohort with 446 melanoma specimens is 33% for class-1 mutation (26% for p.V600E and 6.1% for p.V600K), 3.1% for class-2 mutations, and 4.9% for class-3 mutations (Table 3). Common locations affected by class-3 *BRAF* mutations were codons 594 (7 tumors), 466 (6 tumors) and 467 (4 tumors).

**Table 2:** *BRAF* mutations in 189 melanomas

aa change	cDNA change	Exon	Case No.^a^	Class^b^
p.G464I^c^	c.1390_1391delinsAT	11	1	likely 2
p.G464R	c.1390G > A	11	1	likely 2
p.G466A	c.1397G > C	11	1 (1)	3
p.G466E	c.1397G > A	11	2	3
p.G466 V	c.1397G > T	11	3 (1)	3
p.S467 L	c.1400C > T	11	4 (1)	3
p.G469A	c.1406G > C	11	2	2
p.G469E	c.1406G > A	11	2 (2)	3
p.G469R	c.1405G > A	11	1	2
p.G469 V	c.1406G > T	11	1 (1)	2
p.N581I	c.1742A > T	15	1	3
p.N581 T	c.1742A > C	15	1	likely 3
p.L584F	c.1750C > T	15	1 (1)	UK
p.E586K	c.1756G > A	15	1	UK
p.G593D	c.1778G > A	15	1	UK
p.D594E	c.1782 T > A	15	1 (1)	3
p.D594G	c.1781A > G	15	1 (1)	3
p.D594N	c.1780G > A	15	5 (2)	3
p.G596C	c.1786G > T	15	1	likely 3
p.L597Q	c.1790 T > A	15	2 (1)	2
p.L597R	c.1790 T > G	15	1 (1)	likely 2
p.L597S	c.1789_1790delinsTC	15	1	likely 2
p.T599dup	c.1795_1797dup	15	1	UK
p.V600E	c.1799 T > A^d^	15	117 (44)	1
p.V600K	c.1798_1799delinsAA	15	27 (7)	1
p.V600R	c.1798_1799delinsAG	15	3 (1)	1
p.K601E	c.1801A > G	15	4 (2)	2
p.R603*	c.1807C > T	15	1	UK
p.S605I^c^	c.1814G > T	15	1 (1)	UK
p.S607F	c.1820C > T	15	1	UK

aa change: amino acid change; Case no.: case number. UK: unknown class

^a^Number in the parenthesis indicates case number of *BRAF* mutations previously reported [17]

^b^Likely 2 or 3: other mutations involving the same codon have been categorized as 2 or 3

^c^Not reported in the COSMIC database

^d^Two with c.1799_1800delinsAA

**Table 3:** Incidences of class-1, class-2 and class-3 *BRAF* mutations

	*BRAF*	Class-1	p.V600E	p.V600K	Class-2	Class-3	Unclassified
Uveal (*n* = 2)	0	0	0	0	0	0	0
Mucosal (*n* = 20)	1	0	0	0	0	1	0
Cutaneous (*n* = 286)	132 (46%)	105 (37%)^a^	82 (29%)	20 (7.0%)	10 (3.5%)	14 (4.9%)	3
H/N/UB (*n* = 98)	44 (45%)	28 (29%)	14 (14%)	14 (14%)	5 (5.1%)	10 (10%)	1
non-H/N/UB (*n* = 188)	88 (47%)	77 (41%)^a^	68 (36%)	6 (3.2%)	5 (2.7%)	4 (2.1%)	2
Others (*n* = 138)^b^	56 (41%)	42 (30%)	35 (25%)	7 (5.1%)	4 (2.9%)	7 (5.1%)	4
Total (*n* = 446)	189 (42%)	147 (33%) ^a^	117 (26%)	27 (6.1%)	14 (3.1%)	22 (4.9%)	7 (1.6%)

H/N/UB: Primary tumors at head, neck or upper back

^a^Including 3 tumors with p.V600R

^b^Metastatic melanomas of unknown origin or without information of the primary site, including one tumor with p.S605I and p.V600E within the same allele

**Fig. 2:**
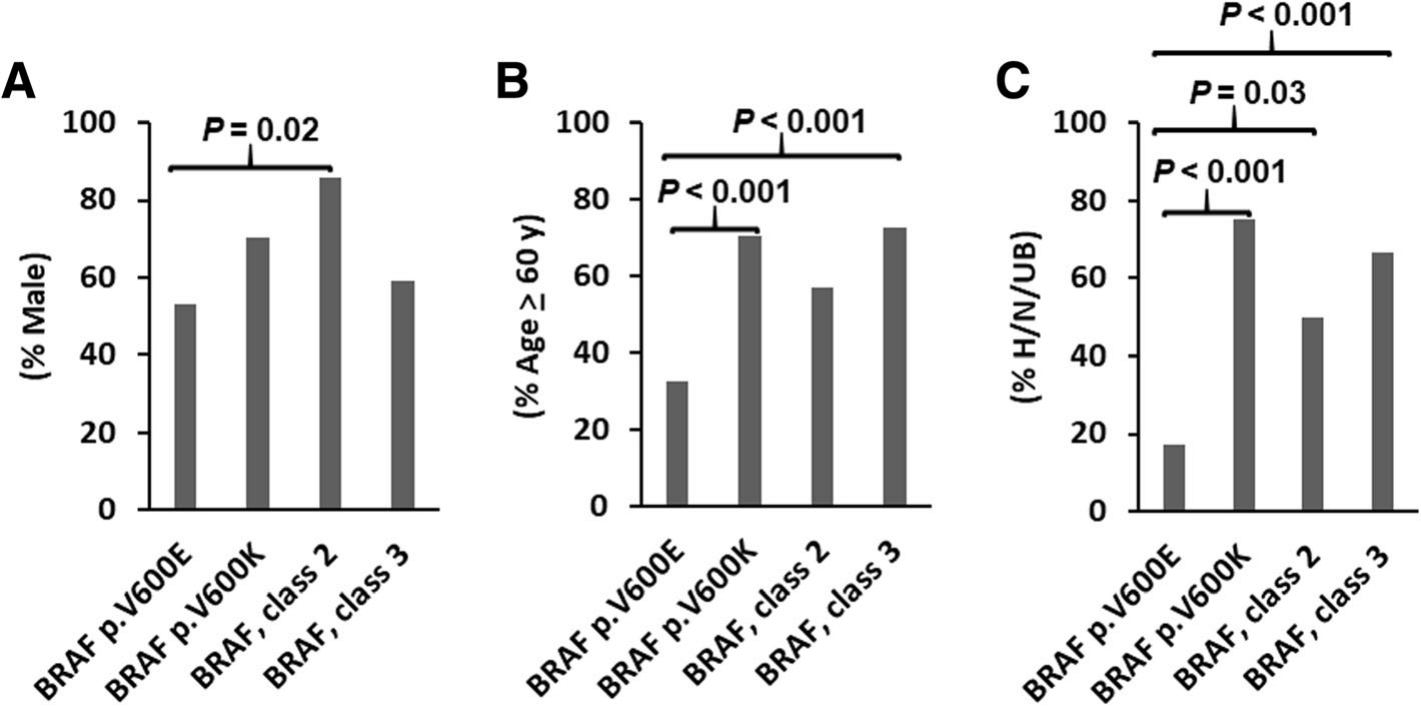
Association of p.V600E (*n* = 117), p.V600K (*n* = 27), class-2 (*n* = 14) and class-3 (*n* = 22) *BRAF* mutations with gender (**a**), age (**b**) and locations of the primary tumor at head, neck and upper back (H/N/UB) (**c**). The primary tumor site was known in 82, 20, 10 and 15 for melanomas with p.V600E, p.V600K, class-2 and class-3 mutations, respectively

We and others have previously shown different clinical characteristics between patients with *BRAF* p.V600E and p.V600K mutation [32–34]. Therefore, *BRAF* class-1 p.V600E and p.V600K mutations were separated for comparison with the class-2 and class-3 mutations. The p.V600K, class-2 and class-3 mutations were associated with male gender and/or old age (> 60 years), as compared to the p.V600E mutation (Fig. 2a and b). In both male and female patient populations with *BRAF* mutations, old age was significantly associated with a higher incidence of p.V600K, class-2 or class-3 mutations and a lower incidence of p.V600E mutation (Additional file 1: Table S2). In old patient and young patient populations, there was no significant association of different *BRAF* mutations with gender, although male patients showed a trend of higher incidence of p.V600K, class-2 or class-3 mutations (*P* = 0.17) and a lower incidence of p.V600E mutation (*P* = 0.07) in the old patient population. The p.V600K, class-2 and class-3 mutations showed a significantly higher incidence of primary tumors located at head, neck and upper back as compared to the p.V600E mutation (75, 50 and 67% vs. 17%, Fig. 2c).

### Association of primary tumors located within the sun exposure areas with the *BRAF* p.V600K mutation and class 3 *BRAF* mutations

The incidence of *BRAF* mutation was similar in cutaneous melanomas with the primary tumor located within or outside the head, neck and upper back areas (45% vs. 47%). However, cutaneous melanomas with the primary tumor located within the head, neck or upper back areas showed a higher incidence of p.V600K mutation (14% vs. 3.2%, *P* = 0.001), class-2 mutations (5.1% vs. 2.7%, *P* = 0.32) and class-3 mutations (10% vs. 2.1%, *P* < 0.01), but a lower incidence of class-1 mutations (29% vs. 41%, *P* = 0.04) and p.V600E mutation (14% vs. 36%, *P* < 0.001), as compared to cutaneous melanomas with the primary tumor located outside the head, neck and upper back areas (Table 3). There was no difference in the incidence of each mutation category between the cutaneous melanomas and metastatic melanomas of unknown origin or without information of the primary site.

### *NRAS*, *KRAS*, *HRAS* and *PIK3CA* mutations

A total of 18 unique *NRAS* mutations were detected in 110 *NRAS*-mutated melanomas, including 2 tumors with 2 *NRAS* mutations. There were 92 (82%) *NRAS* mutations involving codon 61, and 16 (14%) mutations involving codon 12 or codon 13. p.Q61R (38%) and p.Q61K (31%) were two most common *NRAS* mutations. *KRAS* mutations were detected in 12 melanomas. There were 8 mutations involving codon 12 or 13 and one mutation involving codon 61. *HRAS* mutations were detected in 9 melanomas including 2 with p.G13 N (c.37_38delinsAA) resulting from a CC > TT (or GG > AA) alteration, a signature of UV-damage. There were 4 mutations involving codon 12 or codon 13 and 4 mutations involving codon 61. *PIK3CA* mutations were detected in 12 melanomas, including 1 tumor with two *PIK3CA* mutations. p.E545K (38%) was the most common *PIK3CA* mutation. Mutations affecting the 3 most common codons (p.E542, p.E545 and p.H1047) account for only 54% of *PIK3CA* mutations.

### *KIT* mutations

A total of 17 unique *KIT* mutations were detected in 22 melanomas, include one with two *KIT* mutations and one with 3 *KIT* mutations (Additional file 1: Table S3). There were 16 (64%) exon 11 mutations which might be sensitive to imatinib and 5 (20%) exon 17 mutations which are resistant (codon 816) or intermediately responsive (codon 822) to imatinib. p.L576P (28%) was the most common *KIT* mutation.

### Variant allele frequency (VAF)

NGS detected 355 mutations among the 474 specimens. The VAF was 2–5% in 21 (5.9%) of 355 mutations, 2–10% in 47 (13%) mutations, and 2–20% in 87 (25%) mutations (Additional file 1: Table S4). VAF was below the limit of detection of pyrosequencing (approximately 5% VAF) in 4.6 and 4.1% of *BRAF* and *NRAS* mutations, respectively. VAF was below the limit of detection of Sanger sequencing (approximately 10–20% VAF) in 13–28% of *BRAF* mutations, 9–15% of *NRAS* mutations, and 15–38% of *KIT* mutations.

### Mutational profiling of paired specimens

Paired specimens were submitted from 19 patients. Mutational profile was concordant in 17 pairs and discordant in 2 pairs (Table 4). In pair 18, a biopsy specimen taken from the left lower lip showed malignant melanoma with a mitosis rate at 10/mm^2^ and a *BRAF* p.V600E mutation at 11% VAF, consistent with an estimated tumor cellularity at 20–40%. However, the same *BRAF* mutation was not detected in a biopsy specimen with a 60–80% estimated tumor cellularity of malignant melanoma taken from the lower lip one month later. This specimen showed a mitosis rate at 0/mm^2^. In pair 19, no mutations were detected in a resection specimen of right vulva with a diagnosis of melanoma in situ and 20–40% estimated tumor cellularity. However, a resection specimen take from right vulva 39 months later showed malignant melanoma harboring a *KIT* p.N655K mutation at 3.7% VAF in a context of 30–50% estimated tumor cellularity.

**Table 4:** Mutational profiling of 19 patients with paired specimens

Cases	Specimens	Mutations^a^
Same mutation		
Pair 01	Skin, neck (Re)	*NRAS* p.Q61L
	Lymph node, neck (Re)	*NRAS* p.Q61L
Pair 02	Skin, thigh (Re)	*NRAS* p.Q61R
	Soft tissue, thigh (Re)	*NRAS* p.Q61R
Pair 03	Skin, upper arm (Bx)	*NRAS* p.Q61R
	Kidney (FNA)	*NRAS* p.Q61R
Pair 04	Soft tissue, axillar (Re)	*NRAS* p.G13R
	Lymph node, axillar (Bx)	*NRAS* p.G13R
Pair 05	Lymph node, neck (Re)	*BRAF* p.V600K
	Lung (Re)	*BRAF* p.V600K
Pair 06	Soft tissue, upper arm (Re)	*NRAS* p.Q61R
	Soft tissue, scalp (Bx)	*NRAS* p.Q61R
Pair 07	Lymph node, axillar (Re)	*BRAF* p.V600E
	Soft tissue, chest (Re)	*BRAF* p.V600E
Pair 08	Lymph node, groin (Bx)	*BRAF* p.V600E
	Lymph node, neck (FNA)	*BRAF* p.V600E
Pair 09	Skin, scalp (Bx)	No mutation
	Lymph node, neck (FNA)	No mutation
Pair 10	Skin, thigh (Re)	*NRAS* p.Q61R
	Lymph node, iliac (Bx)	*NRAS* p.Q61R
Pair 11	Soft tissue, axillar (Re)	*NRAS* p.Q61R
	Lymph node, axillar (Re)	*NRAS* p.Q61R
Pair 12	Skin, lower leg (Re)	*NRAS* p.Q61L
	Lymoh node, groin (Re)	*NRAS* p.Q61L
Pair 13	Skin, upper back (Bx)	*BRAF* p.V600E
	Soft tissue, upper back (Bx)	*BRAF* p.V600E
Pair 14	Brain (Re)	*NRAS* p.Q61R
	Lymph node, neck (Re)	*NRAS* p.Q61R
Pair 15	Skin, chest (Re)	No mutation
	Lymph node, axillar (Bx)	No mutation
Pair 16	Skin, nose (Re)	*KIT* p.L576
	Liver (Bx)	*KIT* p.L576
Pair 17	Lung (Re)	No mutation
	Lung (Re)	No mutation
Different mutations		
Pair 18	Left lower lip (Bx)	*BRAF* p.V600E
	Lower lip (Bx)	No mutation
Pair 19	Skin, right vulva (Re)	No mutation
	Skin, right vulva (Re)	*KIT* p.N655K

Bx: biopsy; FNA: fine needle aspiration; Re: resection or excision

^a^Mutations in the *BRAF*, *HRAS*, *KIT*, *KRAS*, *NRAS* and *PIK3CA* genes

### Coexisting mutations

Coexisting mutations within *BRAF*, *RAS*, *PIK3CA* and *KIT* genes were observed in 31 (9.5%) of 328 tumors with mutations (Table 5). There were 6 tumors with coexisting mutations of the same gene (1 with 2 *BRAF*, 2 with 2 *NRAS*, 1 with 2 *PIK3CA*, 1 with 2 *KIT*, and 1 with 3 *KIT* mutations), and 26 tumors with coexisting mutations of different genes, including a tumor with 2 *NRAS* and one *PIK3CA* mutation. *BRAF* and *NRAS*-mutated melanomas showed a significantly lower incidence of coexisting mutations of different genes (10 and 8.1%, respectively) as compared to *PIK3CA*, *HRAS* and *KIT*-mutated melanomas (75, 67 and 36%; all *P* < .001).

**Table 5:** Melanomas with coexisting mutations in 31 melanomas^a^

	*BRAF*	*HRAS*	*KRAS*	*NRAS*	*PIK3CA*	*KIT*	CM (%)^b^
*BRAF*	1^c^	3	1	4	5	5	18 (10%)
*HRAS*	3	0	1	0	1	1	6 (67%)
*KRAS*	1	1	0	1	0	0	3 (25%)
*NRAS*	4	0	1	2^d^	2	1	9 (8.1%)
*PIK3CA*	5	1	0	2	1	1	9 (75%)
*KIT*	5	1	0	1	1	2^e^	8 (36%)

^a^Including 29 tumors with 2 mutations and 2 tumors with 3 mutations

^b^Coexisting mutation (CM) within different genes

^c^p.S605I with unknown kinase activity and p.V600E within the same allele

^d^One with *NRAS* p.Q61L and p.Y64D of unknown activating status, and one with *NRAS* p.G13D, p.Q61K and *PIK3CA* p.E542G

^e^One with p.V560A and p.N822Y, and one with p.P573S, p.F681I and p.N822I

### Coexisting *BRAF* and activating *PIK3CA* or *RAS* mutations

Coexisting *BRAF* and activating *RAS* mutations were observed in none of 147 melanomas with a class-1 *BRAF* mutation, 2 (14%) of 14 melanomas with a class-2 *BRAF* mutation, and 5 (23%) of 22 melanomas with a class-3 *BRAF* mutation (Table 6). The incidence of coexisting activating *RAS* mutations was significantly higher in the class-2 and class-3 *BRAF* mutations (*P* < 0.01 and *P* < .001, respectively), as compared to the class-1 *BRAF* mutations. The VAF of *BRAF* mutations were relatively concordant with that of *RAS* mutations, except for one tumor with coexistence of class-2 *BRAF* mutation and *NRAS* mutation. Activating *PIK3CA* mutations affecting codons 542, 545 or 1047 were observed in 2 melanomas with a class-1 *BRAF* mutation and 1 melanoma with a class-3 *BRAF* mutation.

**Table 6:** Coexisting *BRAF* and activating *RAS* or *PIK3CA* mutations

BRAF kinase activity	*BRAF* ^a^	*RAS* or *PIK3CA*^a^
Coexisting *BRAF* and *RAS*^b^		
Class-2 *BRAF* mutation	p.K601E (35%)	*NRAS* p.G13 N (34%)
	p.G464R (5.5%)	*NRAS* p.Q61R (55%)
Class-3 *BRAF* mutation	p.G466A (30%)	*KRAS* p.G12D (49%)
	p.G466 V (19%)	*HRAS* p.G13 N (26%)
	p.S467 L (26%)	*NRAS* p.Q61K (24%)
	p.D594N (47%)	*NRAS* p.G12S (42%)
	p.D594N (59%)	*HRAS* p.Q61K (46%)
Coexisting *BRAF* and *PIK3CA*^c^		
Class-1 *BRAF* mutation	p.V600E (11%)	*PIK3CA* p.545 K (7.5%)
	p.V600K (56%)	*PIK3CA* p.545 K (24%)
Class-3 *BRAF* mutation	p.G466E (17%)	*PIK3CA* p.545 K (3.1%)

^a^Percentage in the parenthesis indicates mutant allele frequency

^b^A case with *BRAF* p.V600E and HRAS p.Q25* was not included

^c^Including only *PIK3CA* mutations affecting codons 542, 545 or 1047

## Discussion

NGS has been clinically validated for mutational profiling of melanomas [18, 19, 35]. We have previously shown a test feasibility of 95% among the first 165 melanoma specimens submitted for NGS testing [36]. This retrospective analysis of 502 melanoma specimens for quality assessment reaffirms the strength of NGS. NGS demonstrates a great analytic sensitivity and broad reportable ranges in clinical mutational profiling. With an analytic sensitivity of 10–20% VAF, Sanger sequencing would have missed 13% or 28% of *BRAF* mutations with less than 10% or 20% VAFs. The analytic sensitivity can be improved to approximately 5% VAF by mutation-specific real time PCR assays, such as cobas *BRAF* mutation test, which was designed to detect only hot spot codon 600 mutations [15, 37]. Eight p.V600E or p.V600K mutations with less than 5% VAF and all non-codon 600 mutations in this series would have been missed by the cobas *BRAF* mutation test.

NGS detected a variety of *BRAF* mutations located outside the reportable ranges of cobas *BRAF* mutation test. These included 43 (9.6%) non-codon 600 *BRAF* mutations detected from 446 melanoma specimens. We have previously categorized these non-codon 600 mutation based on their kinase activity and proposed potential treatment strategy [17]. More recently, *BRAF* mutations are further categorized into 3 classes based on their distinct mechanisms to activate MAPK pathway [22, 23]. BRAF kinase activity are high for class-1 mutants, high to intermediate for class-2 mutants, and impaired or dead for class-3 mutants. Both class-1 and class-2 mutants are RAS-independent, and signal as monomers and dimers, respectively. Class-3 mutants amplify ERK signaling in a RAS-dependent fashion. Therefore, they require coexisting mechanisms to maintain activation of RAS. This is supported by the observation of a significantly higher incidence of coexisting *KRAS*/*NRAS*/*HRAS* mutations in melanomas with class-3 mutations [17, 18, 21, 23]. In this study, coexisting *RAS* mutation was observed in 23% of class-3 mutants, but none of the 147 class-1 mutants when specimens with prior treatment of BRAF inhibitor were excluded. Among the 502 submitted specimens, the only tumor carrying coexisting class-1 *BRAF* mutation and activating *NRAS* mutations was seen in a pleural effusion specimen taken 3 months after combined therapy with BRAF inhibitor and MEK inhibitor. Detection of *BRAF* p.V600E (VAF of 49%), *NRAS* p.Q61K (VAF of 19%) and *NRAS* p.Q61 (VAF of 3.3%) in this pleural effusion specimen are consistent with two *NRAS*-mutated resistant subclones emerging following targeted therapy [38]. Since *NF1* gene is not included in the AmpliSeq Cancer Hotspot Panel (v2), the incidence of coexistence of *NF1* and class-3 *BRAF* mutations is not known in this series [23].

In addition to select patients with melanomas for targeted therapy, mutational status, such as *BRAF* vs. *NRAS* mutation or p.V600E vs. p.V600K *BRAF* mutations may affect clinicopathological characteristics and outcomes [32–34, 39]. p.V600K mutation has been associated with male gender, old age and/or head/neck primary tumor location [32–34]. In a Chinese population examined by Sanger sequencing of exon 15, kinase impaired *BRAF* mutations involving codons 594 and 596 were observed in 7 (3.4%) of 208 mucosal melanomas and 6 (1.1%) of 544 non-mucosal melanomas. Codons 594/596 mutation predicted a good prognosis in this study with a small cohort of kinase impaired *BRAF* mutations [26]. NGS examination using AmpliSeq sequencing panel among 699 advanced melanomas revealed 6% non-codon 600 *BRAF* mutations [18]. Non-V600 mutations are more common in primary tumors of the head and neck.

Categorization in a large cohort of *BRAF*-mutated non-small cell lung cancers based on the new classification system have showed less favorable outcome in patient with class-2 or class-3 *BRAF* mutations [28]. In this study, we applied this new classification system to a cohort of 446 melanoma specimens submitted to a clinical diagnostics laboratory. NGS identified 33% class-1 *BRAF* mutations and 9.6% non-codon 600 mutation, including 3.1% class-2 mutation, 4.9% class-3 mutations and 1.6% unclassified mutations. However, referring of specimens with wild type *BRAF* codon 600 to the laboratory for NGS may have led to a bias toward a lower incidence of class-1 mutation. The current study confirms a previous consensus that p.V600K is more prevalent in old male and primary melanoma of head/neck. This is also true for non-V600 *BRAF* mutations, especially the class-3 mutations. Our findings support that duration of sun damage is a significant contributory factor for both p.V600K and class-3 mutations. A larger cohort of class-2 mutations is needed for further clarification.

Classification of the *BRAF* mutations according to their mechanisms to induce activation of the MAPK pathway also provides rationales for therapeutic strategy in the future [23, 24]. Class-2 and class-3 mutants function as RAS-dependent dimers and RAS-independent dimers respectively [22, 23]. Therefore, melanomas with class-2 or class-3 mutations are not expected to respond to current FDA-approved BRAF inhibitors, which are monomer selective. Case studies have shown benefit of MEK inhibitors in melanoma patients with class-2 mutation [40–42]. Combined therapy with BRAF and MEK inhibitors have shown improved efficacy in patients class-1 *BRAF* mutations, [9, 10] as well as inhibitory effects in cell lines harboring class-2 or class-3 *BRAF* mutations [43, 44]. Recently developed dimer inhibitors, such as αC-IN inhibitor and RAF inhibitor PLX8394 have therapeutic potential against the class-2 and class-3 *BRAF* mutants [45, 46].

Tumor heterogeneity may have important clinical implications. Multiregional analyses to include the precursor lesions have shown that *BRAF* and *NRAS* mutations are early drivers for melanoma tumorigenesis [47]. Therefore, they are expected to be present in the primary tumor and the metastatic tumor. However, evidences of intra-tumor and inter-tumor heterogeneity have been repeatedly reported. Although a high discordance rate of the *BRAF* p.V600E mutation has been reported in 8 (44%) of 18 paired primary and metastatic melanoma specimens, [48] *BRAF* and *NRAS* mutations are generally highly concordant with variation of discordance rates depending on the metastatic sites in larger cohort studies [49, 50]. In this study for quality assessment, discrepancy of *BRAF* and *NRAS* mutations is not identified in 9 pairs of primary and metastatic specimens and 8 pairs of metastatic specimens. Discordance of *BRAF* or *KIT* mutation was seen in two pairs of primary tumors. Tissue identify was confirmed by analysis of 17 single nucleotide polymorphisms within the NGS panel, according to an operation procedure proposed for validation of discordant trunk drivers in patients with multiple lung cancer specimens [51]. Our findings are consistent with a higher discordance rate in patients with multiple primary melanomas or multiple lung nodules [52, 53].

## Conclusion

In this retrospective study for quality assessment, NGS demonstrates a high analytic sensitivity and broad reportable ranges. A variety of recurrent *BRAF* mutations were detected in melanomas in a clinical diagnostics setting. Categorization of 3 classes of *BRAF* mutations according to their mechanisms of signal transduction to activate MAPK pathway showed sun damage could contribute to tumorigenesis of melanomas carrying the *BRAF* p.V600K mutation or class-3 *BRAF* mutations. Further prospective studies are warranted to elucidate the clinical outcomes and benefits of newly developed targeted therapy in melanoma patients carrying each class of *BRAF* mutation.

## Additional file

Additional file 1: **Table S1.** 474 specimens from 457 tumors of 455 patients with melanoma. **Table S2.** Patients stratified according to gender and age. **Table S3.** *KIT* mutations in 22 melanomas. **Table S4.** Variant allele frequency (VAF) detected by next generation sequencing. (DOCX 22 kb)

## Data Availability

Data will be made available upon email request to MTL.

## Abbreviations

BAM: Binary sequence alignment/map
FFPE: Formalin-fixed paraffin-embedded
IGV: Integrative Genomics Viewer
MAPK: Mitogen-activated protein kinase
NGS: Next-generation sequencing
PCR: Polymerase chain reaction
VAF: Variant allele frequency
