# Supplementary material for: Clinical mutational profiling and categorization of BRAF mutations in melanomas using next generation sequencing
Source: BMC Cancer. 2019 Jul 5;19:665. doi: 10.1186/s12885-019-5864-1 (PMC6612071; doi:10.1186/s12885-019-5864-1)
Supplement: Supplementary file 1 — Table S1. 474 specimens from 457 tumors of 455 patients with melanoma. Table S2. Patients stratified according to gender and age. Table S3. KIT mutations in 22 melanomas. Table S4. Variant allele frequency (VAF) detected by next generation sequencing. (DOCX 22 kb) [file 12885_2019_5864_MOESM1_ESM.docx]

**Additional file 1: Table S1.** 474 specimens from 457 tumors of 455 patients with melanoma

| Specimens | Patient (n) | Tumor (n) |
| --- | --- | --- |
| Single specimen | 436 | 436 |
| Primary and metastatic tumors with same mutational status | 9 | 9 |
| Two separate metastatic tumors with same mutational status | 8 | 8 |
| Two primary tumors with different mutational status | 2 | 4 |
| Total | 455 | 457^a^ |

n: case number.

^a^ Including 11 specimens with an estimated tumor cellularity of less than 10% and no *BRAF*, *RAS*, *KIT* and *PIK3CA* mutations detected. These 11 specimens were excluded for analysis.

**Additional file 1: Table S2.** Patients stratified according to gender and age

|  | *BRAF* (n) | p.V600E (n) | p.V600K, class-2 or 3 (n) | Others (n) |
| --- | --- | --- | --- | --- |
| Female |  |  |  |  |
| < 60 year | 46 | 38 (83%) | 8 (17%) | 0 |
| > 60 year | 29 | 17 (59%) | 11 (38%) | 1 (3%) |
| P value |  | 0.02 | 0.04 |  |
|  |  |  |  |  |
| Male |  |  |  |  |
| < 60 year | 56 | 41 (73%) | 12 (21%) | 3 (5%) |
| > 60 year | 58 | 21 (36%) | 32 (55%) | 5 (9%) |
| P value |  | <0.001 | <0.001 |  |
|  |  |  |  |  |
| < 60 year |  |  |  |  |
| female | 46 | 38 (83%) | 8 (17%) | 0 |
| male | 56 | 41 (73%) | 12 (21%) | 3 (5%) |
| P value |  | 0.26 | 0.61 |  |
|  |  |  |  |  |
| > 60 year |  |  |  |  |
| female | 29 | 17 (59%) | 11 (38%) | 1 (3%) |
| male | 58 | 21 (36%) | 32 (55%) | 5 (9%) |
| P value |  | 0.07 | 0.17 |  |

n: case number.

**Additional file 1: Table S3.** *KIT* mutations in 22 melanomas

| aa change | cDNA change | Exon | Specimens. |
| --- | --- | --- | --- |
| p.E33K^a^ | c.97G>A | 2 | 1 |
| p.L57S^a^ | c.170T>C | 2 | 1 |
| p.W557R | c.1669T>C | 11 | 1 |
| p.V559A | c.1676T>C | 11 | 2 |
| p.V559G | C.1676T>G | 11 | 1 |
| p.V560A | c.1679T>C | 11 | 1 |
| p.V560D | c.1679T>A | 11 | 1 |
| p.N564S | c.1691A>G | 11 | 1 |
| p.P573S^a^ | c.1717C>T | 11 | 1 |
| p.Q575_P577del | c.1723_1731del | 11 | 1 |
| p.L576P | c.1727T>C | 11 | 7 |
| p.N655K | c.1965T>A | 13 | 1 |
| p.F681I | c.2041T>A | 14 | 1 |
| p.D816V | c.2447A>T | 17 | 1 |
| p.D816Y | c.2446G>T | 17 | 1 |
| p.N822I | c.2465A>T | 17 | 1 |
| p.N822Y | c.2464A>T | 17 | 2 |

aa change: amino acid change; Case no.: case number.

^a^ Not reported in the COSMIC database.

**Additional file 1: Table S4.** Variant allele frequency (VAF) detected by next generation sequencing

| Mutations | VAF 2-5% | VAF 2-10% | VAF 2-20% |
| --- | --- | --- | --- |
| Total (n = 355) | 21 (5.9%) | 47 (13%) | 87 (25%) |
| *BRAF* (n = 194) | 9 (4.6%)^a^ | 26 (13%) | 54 (28%) |
| *NRAS* (n = 121) | 5 (4.1%) | 11 (9.1%) | 17 (15%) |
| *KIT* (n = 26) | 4 (15%) | 4 (15%) | 10 (38%) |

^a^ 7 with p.V600E, 1 with p.V600K and 1 with p.G466V
